# The association between early regulatory problems and adult peer relationship quality is mediated by the brain's allostatic‐interoceptive system

**DOI:** 10.1111/jcpp.14033

**Published:** 2024-06-25

**Authors:** Saša Zorjan, Dieter Wolke, Nicole Baumann, Christian Sorg, Satja Mulej Bratec

**Affiliations:** ^1^ Department of Psychology, Faculty of Arts University of Maribor Maribor Slovenia; ^2^ Department of Psychology University of Warwick Coventry UK; ^3^ Warwick Medical School University of Warwick Coventry UK; ^4^ Department of Population Health Sciences University of Leicester Leicester UK; ^5^ Turner Institute for Brain and Mental Health, School of Psychology Sciences Monash University Melbourne Vic. Australia; ^6^ Department of Neuroradiology, Klinikum rechts der Isar Technical University of Munich Munich Germany; ^7^ Department of Psychiatry and Psychotherapy, Klinikum rechts der Isar Technical University of Munich Munich Germany; ^8^ TUM‐NIC Neuroimaging Center Technical University of Munich Munich Germany

**Keywords:** Regulatory problems, allostatic interoceptive system, insula, peer relationships, crying, sleeping, feeding, Bavarian Longitudinal Study

## Abstract

**Background:**

Early regulatory problems (RPs), i.e., problems with crying, sleeping, and/or feeding during the first years, increase the risk for avoidant personality traits in adulthood, associated with social withdrawal and anxiety. Even more, RPs are linked with functional alterations in the adult default mode and salience networks, comprising the brain's allostatic‐interoceptive system (AIS) and playing a role in social interactions. We investigated whether RPs assessed in infancy are associated with difficulties in adult peer relationships mediated by functional alterations of the AIS.

**Methods:**

As part of a large case‐controlled prospective study, 42 adults with previous RPs and 70 matched controls (mean age = 28.48, *SD* = 2.65, 51% male) underwent fMRI during rest. The analysis focused on the intrinsic functional connectivity (iFC) of key nodes of the AIS. Peer relationship quality was assessed via a semi‐structured Life Course Interview and the YASR scale. In these same individuals, RPs were assessed at ages 5, 20 and 56 months.

**Results:**

RPs in infancy were associated with lower‐quality peer relationships and enhanced functional connectivity of the AIS nodes in adulthood, with a stronger effect for multiple and persistent RPs compared with transient‐multiple or single‐persistent RPs. Importantly, iFC changes of the dorsal mid insula, a primary interoceptive cortex with frontal and temporal regions, mediated the relationship between early RPs and adult peer relationship quality.

**Conclusions:**

Results indicate long‐lasting social and neural changes associated with early RPs. Our findings further implicate the AIS in both interoceptive and social processes, while indicating the need for early screening of early RPs.

## Introduction

Early regulatory problems (RPs), such as excessive crying (after 3 months of age), feeding problems and/or sleeping difficulties (after 5–6 months of age), are relatively common developmental challenges, occurring in approximately 20% of infants (Hemmi, Wolke, & Schneider, 2011). The occurrence of RPs, particularly of multiple and/or persistent RPs, has long‐term consequences, as it increases the chance of behavioural, emotional and social problems in childhood and even adulthood (Galling et al., 2023; Jaekel et al., 2020; Wolke et al., 2023). In adults, multiple and/or persistent RPs especially and robustly increase the likelihood of avoidant personality traits, which are associated with social withdrawal and anxiety (Bäuml et al., 2019; Wolke et al., 2023). In addition, multiple and/or persistent RPs in infancy are linked with lower social support from peers and friends, as well as no apparent ameliorating effect of peer social support on the development of mood disorders (Jaekel et al., 2023). Taken together, these findings suggest that individuals with multiple and/or persistent RPs in infancy and toddlerhood may exhibit global impairments in social functioning that may continue into adulthood.

Early crying, feeding and sleeping problems can be considered problems of allostatic regulation. Allostasis is a process of predicting and correcting any physiological imbalances before they occur. It is a dynamic process of physiological adaptation to the environment that is directly and closely linked to the regulation of physiological and behavioural responses (Ramsay & Woods, 2014; Sterling, 2012). From a developmental (and evolutionary) perspective, a number of theories posit that social development is rooted in allostasis regulation, thereby intimately connecting the processes of allostasis and social functioning (Atzil, Gao, Fradkin, & Barrett, 2018; Filippetti, 2021; Fotopoulou & Tsakiris, 2017; Zoltowski, Failla, & Cascio, 2022). More specifically, due to human infants' complete social dependency on others for regulating their physiological needs (e.g., feeding, calming) in the first months of life, the development of effective allostasis is inherently social. Importantly, the close interdependency of physiological and behavioural state regulation and social affiliation may persist (albeit less intensively) into adulthood (Atzil et al., 2018; Filippetti, 2021; Fotopoulou & Tsakiris, 2017).

In terms of the underlying neurobiology, recent studies suggest that allostasis regulation is supported by the so‐called allostatic‐interoceptive system (AIS) (Kleckner et al., 2017; Zhang et al., 2023). The AIS is a complex brain system continually interacting with the body and the environment, with the goal of regulating allostasis – i.e., anticipating physiological imbalances to address and amend them ahead of time. For successful allostasis regulation, the AIS relies on interoception – the representation of the physiological state of the inner body (Kleckner et al., 2017; Sennesh et al., 2022). The system is comprised of key interoceptive (i.e., mid and posterior insula) and visceromotor (i.e., anterior insula, anterior and mid cingulate cortex and dorsal amygdala) hubs or nodes that connect with cortical and subcortical regions to support the processes of allostasis and interoception (Kleckner et al., 2017). Considering the anatomical coverage of the system as a whole, it is, to a very high extent, comprised of two very well‐known intrinsic brain networks, the default mode network and the salience network (DMN and SN, respectively) (Kleckner et al., 2017). This is in line with the current view of these two networks as domain‐general, connected to each other and other parts of the brain via “rich club hubs,” and integrating intrinsic and extrinsic information from a number of different brain areas (Heuvel & Heuvel & Sporns, 2011). Critically, we recently showed that early RPs are associated with functional alterations in the adult DMN and SN (Bäuml et al., 2019). Crucially, extensive literature confirms the important roles of DMN and SN in social functioning, for example, in social cognition, social network size, social emotion regulation and maternal bonding (Atzil et al., 2018; Barrett & Satpute, 2013; Bickart, Dickerson, & Barrett, 2014; Xie et al., 2016; Yeshurun, Nguyen, & Hasson, 2021).

Considering the close associations between RPs, allostasis regulation, the allostatic‐interoceptive brain system and social functioning, the current study tested whether multiple and/or persistent RPs in infancy are associated with (a) adult peer relationship quality and (b) functional connectivity of the AIS nodes. We further hypothesised that multiple *and* persistent RPs in infancy would show the strongest associations with both processes compared to the multiple *or* persistent RP subgroups. Finally, we hypothesised that functional connectivity alterations of the AIS due to RPs would mediate the effect of early RPs on adult peer relationships.

## Methods

### Study design and participants

The Bavarian Longitudinal Study (BLS) (Riegel, Ohrt, Wolke, & Österlund, 1995) is a prospective geographically defined birth cohort study of neonatal at‐risk children born in Southern Bavaria, Germany between January 1985 and March 1986 (*N* = 7,505, 10.6% of all live births), who required admission to a children's hospital within 10 days after birth. In addition, 916 healthy infants born at term in the same obstetric hospitals were recruited as controls.

Parents were first approached within 48 hr of the infant's hospital admission. Ethical approval for the studies was granted by the ethics committees of the University of Munich Children's Hospital, the Bavarian Health Council (Landesärztekammer Bayern) and the University Hospital Bonn. Written informed consent was obtained from parents for infant assessments and participants themselves for adult assessments.

**Figure 1:**
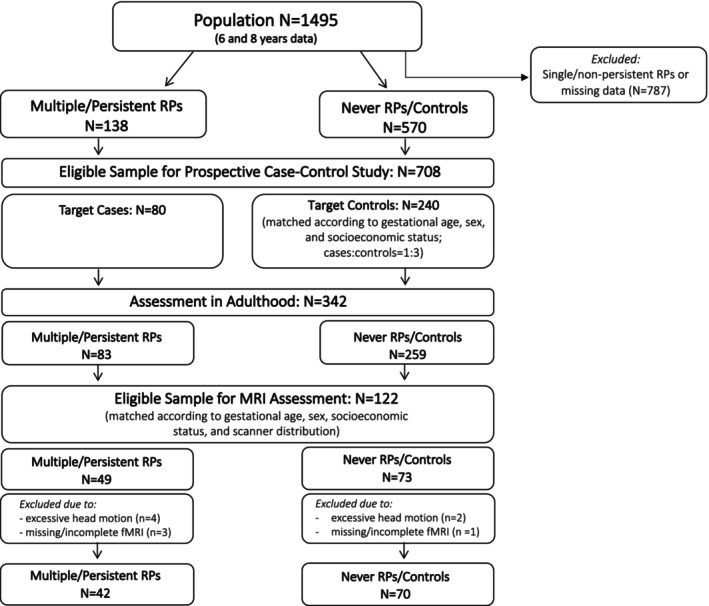
The flow diagram illustrating the original and final study samples. MRI, magnetic resonance imaging; RPs, regulatory problems

After the first phase of the study (birth to 56 months), the sample size was reduced to allow for more intensive psychological and neurological assessments while preserving sufficient statistical power (Jaekel, Baumann, & Wolke, 2013) (see Figure 1). For the prospective case–control follow‐up study, we excluded (a) those participants who at any time had single (e.g., only sleeping problems) or non‐persistent RPs (i.e., RPs present at one or two measurement points) and (b) all cases with any missing data on crying, sleeping, or feeding problems at assessments at 5, 20 and 56 months (*N* = 787). Of the remaining eligible 708 participants (*N* = 138 with multiple or persistent RPs and *N* = 570 without any RPs at any time), we were able to follow up *N* = 83 with multiple or persistent RPs and *N* = 259 without RPs into adulthood (Wolke et al., 2023). These 342 participants were invited for the current study and screened for MR‐related contraindications, resulting in an MRI‐eligible sample of 122 individuals. After additionally excluding 10 datasets based on low‐quality or missing fMRI data, the final sample of the current study included 42 adults with RPs and 70 matched controls aged between 25 and 30 (mean age = 28.48, *SD* = 2.65, 50.9% male) (Figure 1). The two groups (multiple and/or persistent RPs vs. never RPs) were stratified according to gestational age, birth weight, sex, socioeconomic status and scanner type (Table 1; for variable definitions, see Table S1). The final MRI sub‐sample of the current study was highly comparable to the entire adult sample (*N* = 342, including participants without the MRI assessment). For comparison, group demographics of the whole adult sample are shown in Table S2. For details on the history of mental and developmental disorders of the two groups (see Table S3).

**Table 1:** Group demographics

	Multiple/Persistent RPs	Never RPs	*p*
Participants: *n* (%)	*N* = 42 (37.5%)	*N* = 70 (62.5%)	
Age in years: Mean (*SD*)	28.8 (1.6)	28.3 (1.7)	.22
Sex: *n* (%)			.81
Female	22 (52.4%)	35 (50%)	
Male	20 (47.6%)	35 (50%)	
Gestational age in weeks: Mean (*SD*)	37.7 (3.5)	37.2 (3.8)	.65
Birth weight in grams: Mean (*SD*)	2,775.7 (829.3)	2,701.6 (921.2)	.67
Familial socioeconomic status: *n* (%)			.26
High	14 (33.3%)	25 (35.7%)	
Middle	14 (33.3%)	31 (44.3%)	
Low	14 (33.3%)	14 (20%)	

We used *t*‐tests (birth weight), Mann–Whitney *U*‐tests (age, gestational age), or *χ* ^2^‐tests (sex, socioeconomic status). RPs, regulatory problems.

### Measures

#### Regulatory problems

At 5 months of age, paediatricians asked parents about their infant's crying, feeding and sleeping behaviours via a standardised interview (Schmid et al., 2010). At 20 and 56 months, sleeping and eating behaviours were assessed via standardised parental interviews, while eating problems were additionally assessed by neurological examinations of oral motor function. Combining parents' responses and results of the neurological examination, we were able to diagnose the existence of crying, feeding and/or sleeping problems for each infant at each measurement point. Detailed criteria for crying, sleeping and eating problems at each measurement point are shown in Table S4 and have been reported previously (Schmid et al., 2010). The assessments were carried out corrected for premature birth at 5 and 20 months and at the chronological age at 56 months. Only participants with multiple (at least 2 problems at 5 months) and/or persistent RPs (present at all three measurements) were included in the early RPs group.

#### Adult peer relationship quality

Information on the quality of adult peer relationships was obtained using a semi‐structured Life Course Interview (LCI; 10 items) (Wolke, Copeland, Angold, & Costello, 2013) and the YASR scale (Achenbach, 1997) (one item: “I have problems with making or maintaining friendships”). Together all 11 items assess several aspects of social and peer functioning (i.e., having close friends, can exchange thoughts with peers etc.). This type of measure has been used extensively in other longitudinal studies, such as the Great Smokey Mountain study (Copeland, Wolke, Shanahan, & Costello, 2015; Wolke et al., 2013). All items were dichotomised and contributed to the composite score if participants indicated having problems in that area. The score was standardised using the never RPs group as the reference via a scale transformation, such that the mean of the never RPs group became 0 and standard deviation 1. To aid interpretability the score was reverse coded so that lower values indicate a *lower* quality of peer relationships. For information on items included in the composite score (see Table S5).

#### Magnetic resonance imaging

MRI data were acquired by gradient‐echo‐planar sequences at two different sites (Klinikum rechts der Isar, Technical University of Munich and University Hospital Bonn), using the Philips Achieva 3T TX system or the Philips Ingenia 3T system with an 8‐channel SENSE head coil. Active use of psychotropic medication was an exclusion criterion for taking part in the MRI. Resting state data were collected for 11 min from a gradient‐echo echo‐planar sequence (TE = 35 ms, TR = 2,608 ms, flip angle = 90°, FOV = 230 mm^2^, matrix size = 64 × 63, 41 slices, thickness 3.58 and 0 mm inter‐slice gap, reconstructed voxel size = 3.59 × 3.59 × 3.59 mm^3^) resulting in 250 volumes of BOLD fMRI data per subject. A high‐resolution T1‐weighted 3D‐MPRAGE sequence (TI = 1,300 ms, TR = 7.7 ms, TE = 3.9 ms, flip angle = 15°; 180 sagittal slices, FOV = 256 × 256 × 180 mm, reconstruction matrix = 256 × 256; reconstructed voxel size = 1 × 1 × 1 mm^3^) was also acquired. Participants were instructed to keep their eyes closed and not fall asleep during scanning.

Preprocessing of rs‐fMRI data was conducted with FSL (Jenkinson, Beckmann, Behrens, Woolrich, & Smith, 2012) and included realignment, removal of non‐brain tissue, high‐pass temporal filtering (200 s), co‐registration to structural T1 image (Greve & Fischl, 2009), normalisation to the Montreal Neurological Institute space at 2 × 2 × 2 mm resolution (Andersson, Jenkinson, & Smith, 2007) and spatial smoothing with a Gaussian kernel (FWHM = 5 mm). Six participants were excluded due to excessive head movement (defined as a cumulative motion translation or rotation beyond 3 mm or 3° and mean point‐to‐point translation or rotation beyond 0.15 mm or 0.1°) and another 4 due to missing or incomplete rs‐fMRI data (Figure 1). The final sample included 42 individuals with early RPs and 70 controls. The two groups did not differ in head‐motion variables of point‐to‐point rotation or translation or framewise displacement (Dijk, Sabuncu, & Buckner, 2012; Murphy, Bodurka, & Bandettini, 2007).

**Figure 2:**
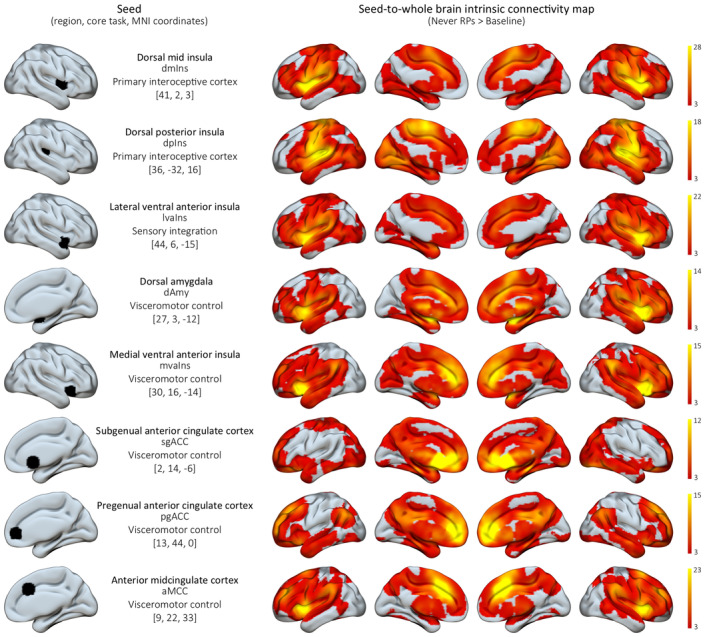
The 8 core AIS regions used as seeds (Kleckner et al., 2017) and corresponding results of the seed‐to‐whole brain analyses (based on the contrast Never RPs > Baseline, FWE‐corrected at *p* < .05 at the cluster level, based on a height threshold of *p* < .001)

To analyse the iFC of the AIS, we followed previous work that identified the AIS (Kleckner et al., 2017). Using SPM 12 (Welcome Trust, London, UK), we carried out a voxel‐wise partial correlation approach to map the iFC between seed time courses of previously defined eight anatomical core nodes of the AIS (Kleckner et al., 2017), i.e., dorsal posterior and dorsal mid insula, medial and lateral ventral anterior insula, dorsal amygdala, subgenual and pregenual ACC, and anterior MCC (see Figure 2) and time‐courses of all other voxels in the brain, regressing out white matter and cerebrospinal fluid signals and six head motion parameters. This resulted in 8 connectivity maps per participant, each representing a different aspect of the AIS via a connectivity profile of a different core node of the AIS (Figure 2).

### Statistical analyses

#### Between‐group differences

To examine group differences in adult peer relationships, a two‐sample *t*‐test was used, as implemented in SPSS, version 26 (IBM Corp., Armonk, NY, USA), with a significance threshold set at *p* = .05. The effect size was estimated as Cohen's *d*.

Group differences in iFC were examined in SPM12 with voxel‐wise two‐sample *t*‐tests, one for each of the 8 connectivity maps from first‐level analysis, controlling for gestational age, sex, scanner type and mean relative head displacement, using a significance threshold of 0.05, FWE cluster‐corrected (based on a height threshold of 0.001).

#### Within‐group differences

To test if persistent, multiple or multiple‐persistent RPs were associated with iFC changes of the AIS to different degrees, we performed one‐way analyses of covariance with three levels (single‐persistent, multiple‐transient, multiple‐persistent RPs), with Bonferroni‐adjusted post‐hoc *t*‐tests. This was realised only for significant group‐different connectivity maps. We controlled for gestational age, sex, scanner type and mean relative head displacement. Effect sizes were estimated as partial eta squared.

#### Brain‐behaviour correlation

To examine whether variance in iFC of the AIS was associated with adult peer relationship quality, averaged iFC values for the group‐different connectivity maps were correlated with the adult peer relationship score, using Spearman's rank correlation and *p* < .05. Spearman's rho was used instead of Pearson's *r* due to its robustness against non‐normality and outliers (Xu, Hou, Hung, & Zou, 2013).

#### Mediation analysis

To test whether the association between RPs and adult peer relationship quality was mediated by the iFC of the AIS, we tested a mediation model using RP group assignment as the independent variable (X), peer relationship quality as the dependent variable (Y) and the iFC of the AIS as the mediator (M). This was realised only if a group difference for a certain AIS node connectivity map was significant and a brain‐behaviour relationship was found, separately for each map, using the mean iFC value of all significant clusters. We used the PROCESS macro (Hayes, 2017), running within SPSS.

## Results

### Infants with RPs have lower‐quality peer relationships in adulthood

We found that infants with RPs on average reported lower quality peer relationships in adulthood (*M* = −0.52, *SD* = 1.38) compared to infants who had no RPs (*M* = 0, *SD* = 1), *t*_110_ = − 2.33, *p* = .02, *d* = 0.45.

### Infants with RPs show enhanced iFC of the AIS nodes, with a scaling effect of RPs

**Figure 3:**
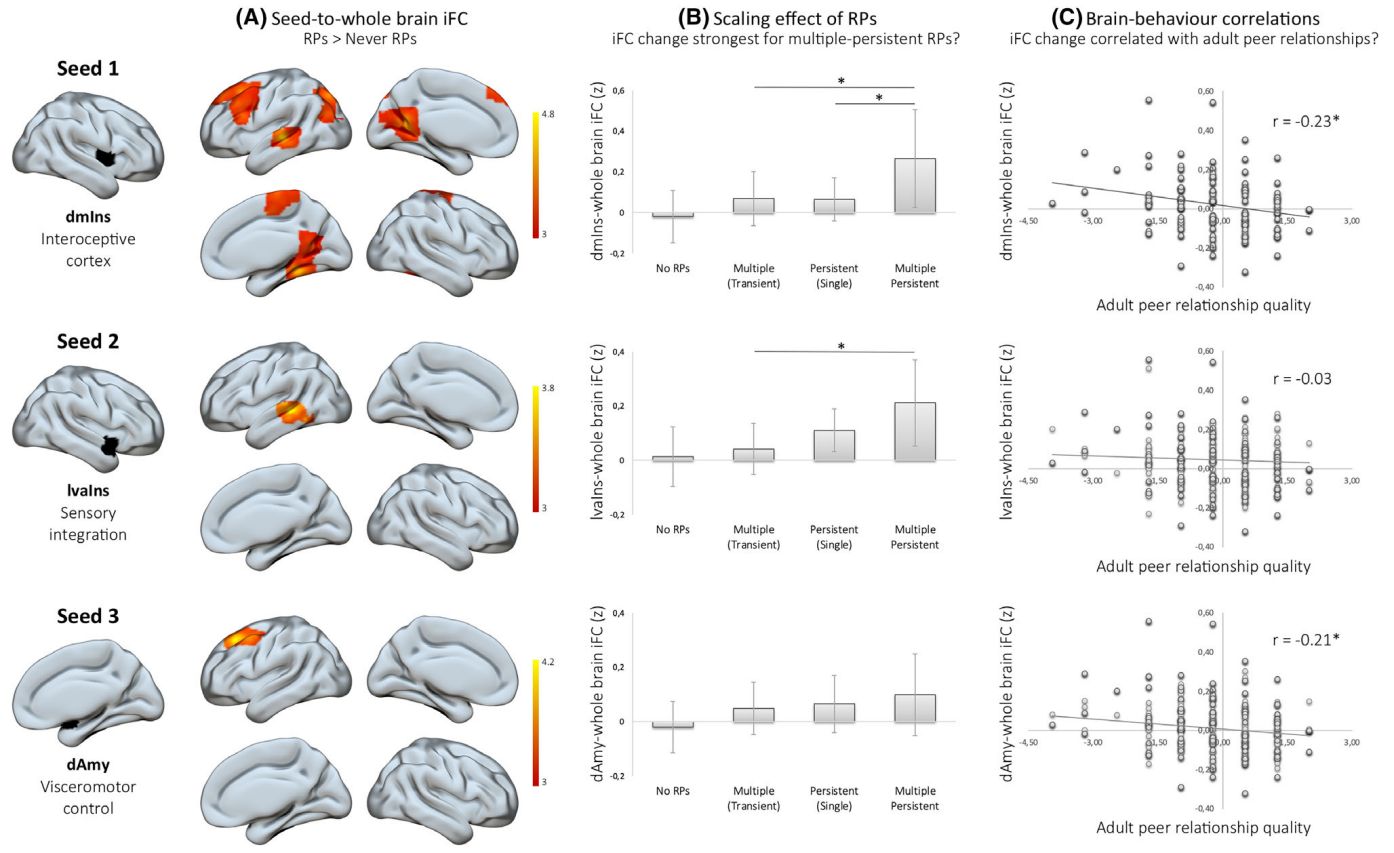
Main results of the study for seeds dmIns, lvaIns and dAmy. (A) Results of paired *t*‐tests RPs > Never RPs, FWE‐corrected (*p* < .05) at the cluster level (height threshold = 0.001; extent thresholds were 219, 222 and 227 for dmIns, lvaIns and dAmy, respectively). (B) For dmIns and lvaIns‐related iFC changes (but not for dAmy), the strength of iFC change depended on RPs severity (*F*_2,36_ = 5.30, *p* = .01, $\eta_{p}^{2}$ = 0.23 for dmIns and *F*_2,36_ = 7.76, *p* = .002, $\eta_{p}^{2}$ = 0.30 for lvaIns). (C) Changes in iFC were associated with peer relationship quality for dmIns (*r* = −.23, *p* = .007) and dAmy (*r* = −.21, *p* = .014), but not lvaIns (*r* = −.03, *p* > .05)

Investigating the association between RPs during infancy and ongoing intrinsic brain connectivity during adulthood, we checked the intrinsic functional connectivity (iFC) of the AIS, using eight core regions of the AIS as seeds (Kleckner et al., 2017). We found that infants with RPs, on average, showed enhanced iFC of the AIS nodes, specifically enhanced iFC of (a) dorsal mid insula (dmIns; primary interoceptive cortex) with the fusiform cortex, middle frontal gyrus, superior temporal gyrus, lateral occipital cortex and precentral gyrus, (b) lateral ventral anterior insula (lvaIns; a sensory integration region) with the middle temporal gyrus, and (c) dorsal amygdala (dAmy; a visceromotor control region) with the superior frontal gyrus (see Figure 3A and Table 2). As shown in Table 2, the dmIns – precentral gyrus cluster and lvaIns – middle temporal gyrus cluster differences did not survive a Bonferroni multiple comparison correction. For dmIns and lvaIns‐related iFC changes (but not for dAmy), we further found that the strength of iFC change was related to the severity of RPs (*F*_2,36_ = 5.30, *p* = .01, $\eta_{p}^{2}$ = 0.23 for dmIns and *F*_2,36_ = 7.76, *p* = .002, $\eta_{p}^{2}$ = 0.30 for lvaIns), such that the stronger increases in iFC were seen for the multiple‐persistent subgroup for both the dmIns (multiple‐persistent vs. multiple: *p* = .015, vs. persistent: *p* = .011, Figure 1A) and the lvaIns result (multiple‐persistent vs. multiple: *p* = .001, vs. persistent: *p* = .059) (Figure 3B).

**Table 2:** Group differences in seed‐to‐whole brain iFC

Region	Cluster size	Peak MNI coordinates			Peak *z*	*p*
		*x*	*y*	*z*		
dmIns‐to‐whole brain iFC (RPs > Never RPs)						
Precuneus cortex, temporal occipital fusiform cortex	1,476	26	−42	−14	4.37	.000
Superior frontal gyrus, frontal pole	609	−30	16	44	4.35	.000
Middle frontal gyrus, inferior frontal gyrus	571	−48	28	40	4.63	.000
Middle temporal gyrus, superior temporal gyrus	396	−56	−24	−2	4.83	.004
Lateral superior occipital cortex	358	−26	−78	46	4.11	.006
Precentral gyrus, postcentral gyrus^a^	237	12	−14	54	3.98	.029
dpIns‐to‐whole brain iFC (RPs > Never RPs)						
No significant clusters						
lvaIns‐to‐whole brain iFC (RPs > Never RPs)						
Middle temporal gyrus, superior temporal gyrus^a^	226	−56	−36	0	3.83	.047
dAmy‐to‐whole brain iFC (RPs > Never RPs)						
Superior frontal gyrus, middle frontal gyrus	545	−22	28	48	4.17	.001
mvaIns‐to‐whole brain iFC (RPs > Never RPs)						
No significant clusters						
sgACC‐to‐whole brain iFC (RPs > Never RPs)						
No significant clusters						
pgACC‐to‐whole brain iFC (RPs > Never RPs)						
No significant clusters						
aMCC‐to‐whole brain iFC (RPs > Never RPs)						
No significant clusters						

Based on paired *t*‐tests RPs > Never RPs for seed‐to‐whole brain iFC analyses, FWE‐corrected (*p* < .05) at the cluster level, based on a height threshold of *p* < .001 (corresponding to extent thresholds 219, 220, 222, 227, 205, 195, 198, 241, for dmIns, dpIns, lvaIns, dAmy, mvaIns, sgACC, pgACC, aMCC, respectively). Anatomical regions were identified with the SPM Anatomy Toolbox (Eickhoff et al., 2005); shown are the top two regions for each cluster (assignment based on maximum probability).

^a^ Did not survive a Bonferroni multiple comparison correction of *p* = .05/8 = .0063.

### Changes in dmIns and dAmy‐related iFC were associated with peer relationship quality

Focusing on the linear brain‐behaviour association, we found a statistically significant negative correlation between peer relationship quality and increased iFC of dmIns (*r* = −.23, *p* = .007) and dAmy (*r* = −.21, *p* = .014), but not lvaIns (*r* = −.03, *p* > .05) (Figure 3C).

### IFC changes of the primary interoceptive cortex (dmIns) mediated the relationship between RPs and peer relationship quality

**Figure 4:**
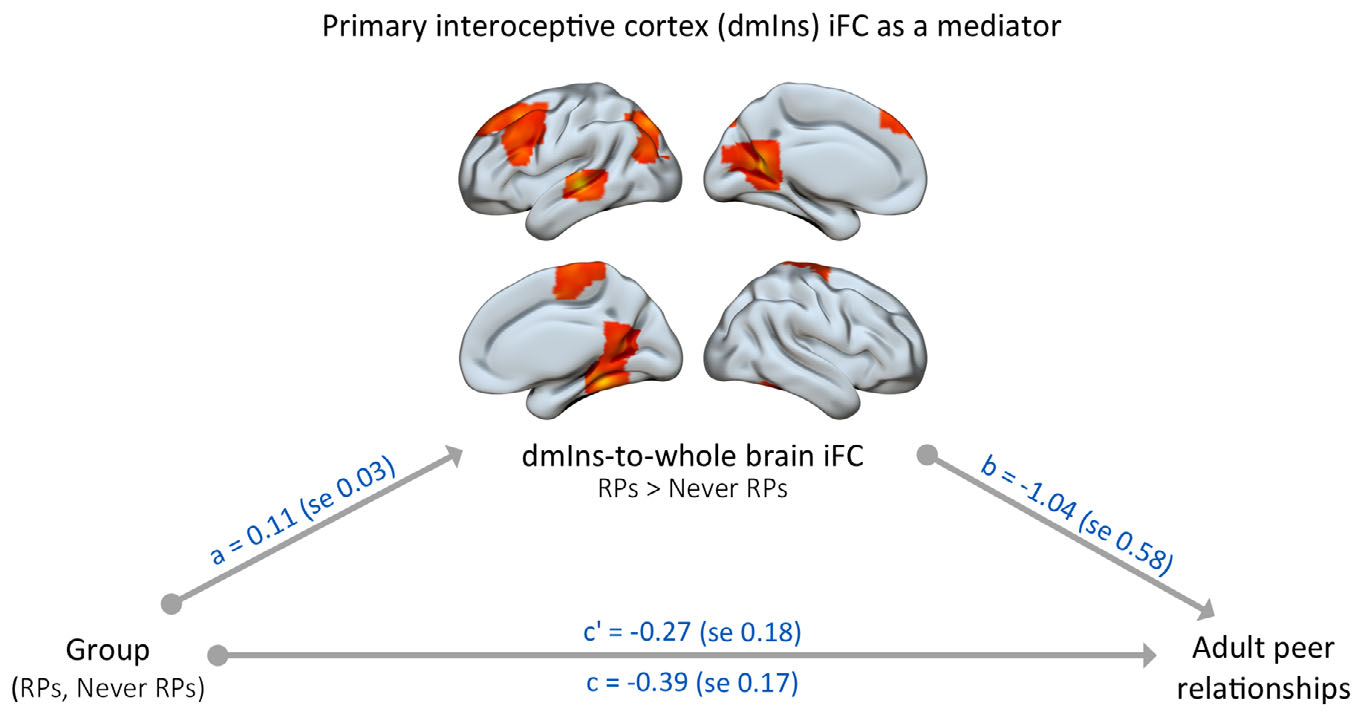
IFC changes of the primary interoceptive cortex (dmIns) fully mediated the relationship between RPs and adult peer relationship quality. The indirect effect of RPs on peer relationship quality through an increase in dmIns iFC was significantly different from zero (95% bootstrapped confidence interval = [−.275, −.002])

The mediation analysis, testing dmIns‐to‐whole brain iFC as a mediator between the history of RPs and adult peer relationships, showed that the significant total effect between RP group and peer relationship quality (*c* = −0.39, *t* = −2.33, *p* = .022, *R* ^2^ = .047) became non‐significant after controlling for mean dmIns iFC values (direct effect *c*' = −0.27, *t* = −1.51, *p* = .134), indicating a full mediation (Figure 4). Confirming the mediation effect itself, the indirect effect of RPs on peer relationship quality via an increase in dmIns iFC was significantly different from zero (95% bootstrapped confidence interval = [−.275, −.002]). Together, the variables RP group and dmIns iFC explained a total of 7.4% of the variance in adult peer relationship quality. In contrast, despite the significant correlation (Figure 3C), dAmy‐to‐whole brain iFC was not a significant mediator between the history of RPs and adult peer relationships.

## Discussion

This study examined associations between early multiple and/or persistent RPs and (a) peer relationships in adulthood and (b) alterations in iFC of the adult AIS. We found that individuals with early RPs had lower‐quality adult peer relationships and exhibited higher iFC of specific core nodes of the AIS in adulthood. In addition, there was a scaling effect, with the multiple *and* persistent RPs sub‐group showing relatively larger iFC changes. Importantly, AIS iFC changes of the primary interoceptive cortex (the dorsal mid insula) with frontal and temporal clusters mediated the relationship between the occurrence of multiple and/or persistent early RPs and adult peer relationship quality.

Results demonstrated lower quality peer relationships, on average, in adulthood for infants with multiple and/or persistent RPs compared with the never RPs group. This is in line with previous studies showing that early RPs are associated with avoidant personality traits (Bäuml et al., 2019; Wolke et al., 2023) and lower peer support (Jaekel et al., 2023) in adulthood. It is further compatible with the notion that regulating bodily functions such as crying, sleeping and feeding is inherently connected with the process of establishing effective social interactions, as the bodily regulation during the first year(s) of life occurs within a close social dyad between the infant and the caregiver (Atzil et al., 2018; Filippetti, 2021; Fotopoulou & Tsakiris, 2017; Zoltowski et al., 2022). Our results are also in line with the developmental literature on the bidirectional nature of parent–child interactions and its impact on a child's developmental trajectory. For example, caregiver‐child reciprocity has been linked to enhanced social competence and self‐regulation in children, especially in those with higher levels of negative emotionality who benefit significantly from responsive relationships (Feldman & Masalha, 2010; Kim & Kochanska, 2012). Extending this to the allostasis literature, the social dyad thus encourages the acquisition of behaviours and concepts necessary for social affiliation via the process of allostasis, which is dependent upon responsive caregiving in early life (Atzil et al., 2018; Atzil & Gendron, 2017; Filippetti, 2021) Consistent with this perspective, optimal maternal care is associated with enhanced social development and physiological regulation during childhood and adolescence (Feldman, 2015).

We further found that early RPs were associated with enhanced iFC of the AIS (Figure 3A). In detail, out of the 8 core AIS nodes (see Figure 2) (Kleckner et al., 2017), occurrence of RPs affected iFC of 3 of the nodes (Figure 3A). (a) The primary interoceptive cortex – the dorsal mid insula seed – showed the most widespread effect of increased iFC, with the fusiform cortex, middle frontal gyrus, superior temporal gyrus, lateral occipital cortex and precentral gyrus. Increased iFC for the RPs group compared to the never RPs group was also found (b) between the lateral ventral anterior insula node and the middle temporal gyrus, and (c) between the dorsal amygdala node and the superior frontal gyrus. No effect of RPs on the iFC of the AIS was found for the remaining 5 out of 8 AIS seeds. Taking multiple comparison correction into account, RPs most notably affected intrinsic connectivity of the dorsal mid insula and the dorsal amygdala. The first is a primary interoceptive cortex that receives interoceptive predictions from the dorsal amygdala, anterior insula and cingulate, as well as ascending visceromotor inputs from the body. It integrates these signals to form prediction errors, which update subsequent interoceptive prediction signals, supporting allostasis (Craig, 2003; Kleckner et al., 2017). The second is a subcortical visceromotor region, sending interoceptive predictions (and receiving prediction errors) to the primary interoceptive cortex, as well as sending visceromotor predictions (and receiving prediction errors) to the hypothalamus and brainstem nuclei (Kleckner et al., 2017; Mufson, Mesulam, & Pandya, 1981).

Interestingly, we found increased connectivity of AIS nodes, while a previous study found reduced iFC of the DMN and SN (Bäuml et al., 2019), the two networks that together significantly overlap with the AIS. We directly tested the iFC of key AIS regions via seed‐based whole‐brain connectivity analysis (Kleckner et al., 2017), while the previous study (a) used independent component analysis, with each identified network only covering core areas of DMN and SN, as well as (b) tested the effects of RPs on each network separately, rather than on a joint system for interoception and allostasis. A large‐scale account of whole‐brain iFC alterations in autism spectrum disorders, characterised by both allostatic dysregulation and social deficits, revealed both significant decreases as well as increases in iFC, the latter focused on subcortical brain areas (Martino et al., 2014). In line with this, our results demonstrate increased connectivity of key AIS regions, perhaps reflecting higher effort or more imprecise interoceptive and/or visceromotor predictions, requiring heightened communication of prediction errors.

Our results further show that the strength of iFC change was dependent on the severity of RPs. In detail, for the dorsal mid insula and the lateral ventral anterior insula seeds, the strongest increases of iFC were seen for the multiple and persistent RPs sub‐group (Figure 3B). A similar scaling effect for DMN connectivity was found in a previous study (Bäuml et al., 2019), implying that having longer‐lasting and multiple RPs has a relatively higher impact on the AIS, suggesting that individuals with both multiple and persistent RPs are the most vulnerable to AIS changes.

Importantly, the current study demonstrated an association between the RP‐related changes in AIS iFC, specifically the dorsal mid insula and the dorsal amygdala iFC, and the quality of peer relationships in adulthood, such that the stronger the iFC effect, the lower the peer relationship quality (Figure 3C). Testing the specificity of the association, additional control correlation analysis demonstrated no similar relationship between the dorsal mid insula or the dorsal amygdala iFC and anxiety or depression scores derived from the YASR (all *p* > .1). This result is consistent with our hypothesis and in line with previous research, whereby iFC changes in the DMN due to RPs were related to avoidant personality traits (Bäuml et al., 2019). Even more crucially, the effect of early RPs on adult peer relationship quality was fully mediated by the RP‐related iFC changes, specifically the iFC between the dorsal mid insula and the fusiform cortex, middle frontal gyrus, superior temporal gyrus, lateral occipital cortex and precentral gyrus (Figure 4). This indicates that alterations of AIS iFC might contribute to lower quality peer relationships in adulthood, and highlights the relevance of the primary interoceptive cortex as a crucial hub within the AIS, as no mediation effect was found for the lateral ventral anterior insula or the dorsal amygdala.

The links between early body regulation, the AIS and adult social relationships can be clarified by developmental accounts of allostasis and social affiliation (Atzil et al., 2018; Filippetti, 2021; Fotopoulou & Tsakiris, 2017; Zoltowski et al., 2022). According to these accounts, social development is intimately connected with allostasis and interoception, as the caregiver helps the infant regulate their bodily functions. As such, both problems with interoception/ allostasis regulation, as well as insensitive caregiving, can impede typical social development (Djerassi, Ophir, & Atzil, 2021). On the one hand, the caregiver's inability to effectively regulate the infant reduces the rewarding value of the social interaction, causing reduced motivation for social proximity. On the other hand, compromised signals for interoception and regulation will require higher effort of the caregiver to effectively attend to the infant's needs and assist in behaviour regulation. Consequently, it may impact attachment relationships and the future self‐regulation of the child (Bilgin & Wolke, 2017, 2020). Furthermore, social care would typically provide statistical regularity of the allostasis‐related reinforcement, fuelling social learning. Disrupted interoception, however, would interfere with social learning, possibly negatively influencing social abilities in the long‐term (Atzil et al., 2018; Djerassi et al., 2021).

Understanding the neurobiological mechanisms of RPs and their long‐term effects may allow for more effective interventions. Maternal sensitivity and touch can affect the resting‐state connectivity of AIS regions (Brauer, Xiao, Poulain, Friederici, & Schirmer, 2016; Chajes, Stern, Kelsey, & Grossmann, 2022), social reappraisal can reduce negative feelings by recruiting key AIS regions (Xie et al., 2016), and the administration of oxytocin affects the activity and connectivity of the insula (Nomi, Molnar‐Szakacs, & Uddin, 2019). Early targeted interventions could be explored by future studies.

A notable strength of the current study is its prospective longitudinal design, following the same individuals from birth to adulthood and measuring RPs before relationships with peers were even established. The careful assessment of multiple variables (e.g., gestational age, sex, socioeconomic status) further allowed for the risk mitigation of confounding variables. Limitations are the smaller MRI sub‐sample that may selectively include mentally and cognitively “fitter” individuals willing to undergo scanning. A more representative sample could potentially show stronger/further differences in AIS connectivity. A further limitation is the lack of fMRI data from infancy and childhood, which would help illuminate the root cause, interaction and trajectory of RPs and (atypical) AIS development. As such, the current data cannot conclusively confirm the direction of the effects, such that, in theory, RPs could have instead negatively influenced social relationships, which affected connectivity of the AIS. We also only focused on the impact of RPs on AIS connectivity and social peer relationships, while other factors could also be of interest, such as parental sensitivity (Breeman et al., 2018; Jaekel et al., 2020). Furthermore, the study did not measure potential allostatic problems in adulthood to be able to relate them with RPs in infancy and/or changes in the AIS in adulthood. Lastly, the hypotheses of the study were only pre‐registered internally and not publicly.

In conclusion, this study indicates that early RPs are associated with changes in AIS connectivity and peer relationship problems some 25 years later. Multiple and persistent RPs have long term adverse effects on the brain and social relationships and should be considered as targets of early treatment.

**Key points**

- Early problems with crying, sleeping and/or feeding (i.e., regulatory problems, RPs) increase the risk of avoidant personality traits via connectivity changes of the default mode network, a crucial part of the allostatic‐interoceptive system (AIS).
- We found that infants with RPs had lower‐quality peer relationships in adulthood, mediated by enhanced connectivity of the AIS.
- Our findings suggest that early RPs negatively impact adult social relationships via impairments of the AIS.
- Results indicate long‐lasting social and neural changes associated with early RPs and highlight relevant targets for potential intervention strategies.

## Supporting information


**Table S1** Definitions of potential confounding variables.
**Table S2.** Group demographics of the whole sample (including participants without MRI data).
**Table S3.** History of mental and developmental disorders.
**Table S4.** Definition of crying, feeding, and sleeping problems at 5, 20, and 56 months, including assessment mode (Schmid, Schreier, Meyer, & Wolke, 2010).
**Table S5.** Items for the composite score of adult peer relationships quality, including source questionnaires, definition, and scoring.

## Data Availability

The data that support the findings of this study are available on request from the corresponding author. The data are not publicly available due to privacy or ethical restrictions.
