# Supplementary material for: The association between early regulatory problems and adult peer relationship quality is mediated by the brain's allostatic‐interoceptive system
Source: J Child Psychol Psychiatry. 2024 Jun 25;66(1):75–84. doi: 10.1111/jcpp.14033 (PMC11652421; doi:10.1111/jcpp.14033)
Supplement: Supplementary file 1 — Table S1 Definitions of potential confounding variables. Table S2. Group demographics of the whole sample (including participants without MRI data). Table S3. History of mental and developmental disorders. Table S4. Definition of crying, feeding, and sleeping problems at 5, 20, and 56 months, including assessment mode (Schmid, Schreier, Meyer, & Wolke, 2010). Table S5. Items for the composite score of adult peer relationships quality, including source questionnaires, definition, and scoring. [file JCPP-66-75-s001.docx]

***Supporting Information***

**The association between early regulatory problems and adult peer relationship quality is mediated by the brain’s allostatic-interoceptive system**

Saša Zorjan, Dieter Wolke, Nicole Baumann, Christian Sorg, Satja Mulej Bratec*

*Correspondence: [satja.mulej@um.si](mailto:satja.mulej@um.si)

**Table S1.** Definitions of potential confounding variables.

**Table S2.** Group demographics of the whole sample (including participants without MRI data).

**Table S3.** History of mental and developmental disorders.

**Table S4.** Definition of crying, feeding, and sleeping problems at 5, 20 and 56 months, including assessment mode (Schmid et al., 2010).

**Table S5.** Items for the composite score of adult peer relationships quality, including source questionnaires, definition and scoring.

**Table S1.** Definitions of potential confounding variables.

| Potential confounding variable | Definition |
| --- | --- |
| Gestational age | Determined from maternal reports of the last menstrual period and ultrasounds during pregnancy. |
| Sex | Biologically determined. |
| Familial socio-economic status (SES) | Collected via structured parental interviews and computed as a weighted composite score. Determined from the occupation of the head of each family together with the highest educational qualification held by either parent. Divided into three categories of low, medium and high SES. |
| Scanner type | Data acquisition in Bonn and Munich switched from Philips Achieva 3T TX system (Achieva, Philips, the Netherlands) to Philips Ingenia 3T system, consistently using an 8-channel SENSE head coil. Final allocation of participants: Bonn 1: 9 participants, Bonn 2: 5 participants, Munich 1: 33 participants, Munich 2: 75 participants. |

**Table S2**. Group demographics of the whole sample (including participants without MRI data).

|  | **Multiple/Persistent RPs** | | **Never RPs** | ***p*** |
| --- | --- | --- | --- | --- |
| Participants: *n* (%) | N = 83 (24.3 %) | N = 259 (75.7 %) | |  |
| Age in years: Mean (SD) | 28.14 (1.85) | 27.44 (1.81) | | .002 |
| Sex: *n* (%) |  |  | | .71 |
| Female | 43 (51.8 %) | 128 (49.4 %) | |  |
| Male | 40 (48.2 %) | 131 (50.6 %) | |  |
| Gestational age in weeks: Mean (SD) | 36.71 (4.39) | 36.73 (4.17) | | .97 |
| Birth weight in grams: Mean (SD) | 2611 (951) | 2705 (960) | | .44 |
| Familial socioeconomic status: *n* (%) |  |  | | .83 |
| High | 26 (31.3 %) | 81 (31.3 %) | |  |
| Middle | 33 (39.8 %) | 111 (42.9 %) | |  |
| Low | 24 (28.9 %) | 67 (25.9 %) | |  |

Note. We used χ2 tests for sex and socioeconomic status and *t* tests for the rest of the variables. RPs – regulatory problems.

**Table S3.** History of mental and developmental disorders.

| **Diagnosis** | **Multiple/Persistent RPs** | | **Never RPs** | ***p*** |
| --- | --- | --- | --- | --- |
| **ADHD** |  |  | |  |
| At 6 years | 8 (19.0%) | 8 (11.4%) | | 0.2 |
| At 8 years | 10 (23.8%) | 6 (8.6%) | | 0.03 |
| **Depression** |  |  | |  |
| At 6 years | 0 | 0 | | n/a |
| At 8 years | 0 | 1 (2.6%) | | 0.72 |
| **Anxiety** |  |  | |  |
| At 6 years | 2 (13.3%) | 6 (15.0%) | | 0.62 |
| At 8 years | 1 (6.7%) | 0 | | 0.28 |

Note. RPs – regulatory problems.

**Table S4.** Definition of crying, feeding, and sleeping problems at 5, 20 and 56 months, including assessment mode (Schmid, Schreier, Meyer, & Wolke, 2010).

| **Regulatory problems** | **Definition** | **Assessment Mode** |
| --- | --- | --- |
| **5 months of age** |  |  |
| Crying problems:  (diagnosed if ≥ 1 of 4 criteria were met) | 1. Cry duration: ≥ 2 hours per day. AND/OR | PI |
|  | 2. Cry amount: above average. AND/OR | PI |
|  | 3. Infant is usually difficult to soothe. AND/OR | PI |
|  | 4. Infant is constantly irritable. | PI |
| Feeding problems:  (diagnosed if ≥ 1 of 3 criteria were met) | 1. Infant does not eat and drink well. AND/OR | PI |
|  | 2. Formerly and currently problems with vomiting. AND/OR | PI |
|  | 3. Disordered oral-motor functioning, i.e., problems with sucking / swallowing, disordered mouth / tongue movement. | PI |
| Sleeping problems:  (diagnosed if ≥ 1 of 2 criteria were met) | 1. Infant wakes up ≥ 2 times per night. AND/OR | PI |
|  | 2. Infant wakes up for ≥ 15 minutes at night. | PI |
| **20 months of age** |  |  |
| Eating problems:  (diagnosed if ≥ 1 of 3 criteria were met) | 1. Occurrence of eating problems. AND/OR | PI |
|  | 2. Problems with chewing, swallowing, or not accepting solid food. AND/OR | NE |
|  | 3. Oral-motor dysfunction, i.e., uncoordinated movements, not harmonic. | NE |
| Sleeping problems: | Occurrence of sleeping problems. | PI |
| **56 months of age** |  |  |
| Eating problems:  (diagnosed if ≥ 1 of 2 criteria were met) | 1. Eating problems/problems with food intake. AND/OR | PI |
|  | 2. Neurological/behavioural dysfunction (motor problems, loss of appetite, refusal to eat, or other problems). | NE |
| Sleeping problems:  (diagnosed if ≥ 2 of 4 criteria were met) | 1. Sleeps through less than three nights per week. | PI |
|  | 2. Needs more than 30 minutes to fall asleep. | PI |
|  | 3. Only falls asleep when parents are around. | PI |
|  | 4. Regularly sleeps in parents’ bed. | PI |

Note. PI = Standardized parental interview; NE = Neurological examination by paediatrician.

**Table S5.** Items for the composite score of adult peer relationships quality, including source questionnaires, definition and scoring.

| **Item** | **Questionnaire** | **Definition** | **Scoring** |
| --- | --- | --- | --- |
| No best friend | Life Course Interview | Had no best friend post school. | 1 |
| Trouble making and keeping friends | YASR | Having somewhat or very much trouble making or keeping friends. | 1 |
|  | Life Course Interview | Had none or only to 2 or 3 friends post school. | 1 |
|  |  | Had no circle of friends post school. | 1 |
| No support | Life Course Interview | No exchange of thoughts and feelings with friends. | 1 |
|  |  | Friends are not supportive regarding career. | 1 |
|  |  | Friends could not be asked for help when in need of care. | 1 |
| Conflict | Life Course Interview | Having difficulties hearing uncomfortable truths from friends. | 1 |
|  |  | Having stressful conflicts with friends. | 1 |
| Socialising | Life Course Interview | Not involved in social activities (e.g. dancing, music, scouts) for more than a year post school. | 1 |
|  |  | Never gone out to bars or clubs post school. | 1 |
| **Total score** |  | | **11** |

Note. For analysis, the total score was standardised on the never RPs group and computed into a negative score, such that higher scores indicated better peer relationships.

**References:**

Schmid, G., Schreier, A., Meyer, R., & Wolke, D. (2010). A prospective study on the persistence of infant crying, sleeping and feeding problems and preschool behaviour. *Acta Pædiatrica*, *99*(2), 286–290.
